# Complete genome sequence of native *Bacillus cereus* strains isolated from intestinal tract of the crab *Ucides* sp.

**DOI:** 10.1016/j.dib.2017.11.049

**Published:** 2017-11-20

**Authors:** João Costa Filho, Sérgio Jorge, Frederico Schmitt Kremer, Natasha Rodrigues de Oliveira, Vinicius Farias Campos, Luciano da Silva Pinto, Odir Antônio Dellagostin, Rubens Galdino Feijó, Francisca Gleire Rodrigues de Menezes, Oscarina Viana de Sousa, Rodrigo Maggioni, Luis Fernando Marins

**Affiliations:** a Federal University of Rio Grande, Institute of Biological Sciences, Rio Grande, RS, Brazil; b Federal University of Pelotas, Center for Technology Development, Biotechnology Unity, Pelotas, RS, Brazil; c Federal Institute of Education, Science and Technology of Ceará, Aracaú, CE, Brazil; d Federal University of Ceará, Institute of Marine Sciences, Fortaleza, CE, Brazil

**Keywords:** Gram positive bacteria, Genome sequence, *Bacillus*

## Abstract

*Bacillus cereus* is a gram positive bacterium with sporulation capacity. Here, we report the complete genome sequence of two native *B. cereus* strains (#25 and #29) isolated from intestinal tract of the crab *Ucides* sp. from Pacoti River in the State of Ceará, Brazil. The findings of this study might increase the molecular information for *Bacillus* strains. The data can be used in comparative analyses, origin and distribution, as well support for genetic engineering.

**Table:** 

**Specifications Table**	

Subject area	Biology
More specific subject area	Microbiology and genomics.
Type of data	Table, text file, image and figure.
How data was acquired	Scanning Electron Microscope (JEOL JSM - 6610 LV), Ion Torrent PGM system and bioinformatics applications.
Data format	Analyzed
Experimental factors	Isolation and characterization of native strains #25 and #29. Genomic DNA extraction and sequencing procedures.
Experimental features	Sequencing using Ion Torrent PGM (Life Technologies, Saint Aubin, France), FASTQ (bamToFastq program from the BedTools package), Phred score (Q) using the fastq_quality_filter script from the fastx-toolkit package (http://hannonlab.cshl.edu/fastx_toolkit/). *De novo* genome assemblies using MIRA (http://mira-assembler.sourceforge.net/), SPAdes and Newbler (http://www.roche.com/), consensus assembly was generated using CISA. Contigs was ordered with the program CAR, using the genome of *Bacillus cereus* strain 03BB102 (GenBank: CP009318.1) as reference. Annotation was performed using Genix and Artemis. A BLAST search was performed (https://www.ncbi.nlm.nih.gov/BLAST/). Chromosome features were drawing using DNAPlotter.
Data source location	The native strains #25 and #29 were isolated from intestinal tract of the crab *Ucides* sp., from Pacoti River in Fortaleza, State of Ceará, Brazil. Latitude: 03° 43′ 02″ S and Longitude: 38° 32′ 35″ W.
Data accessibility	The complete genome sequence of native *B. cereus* strains #25 and #29 were deposited in GenBank - NCBI under the accession number CP020803.1 and CP020804.1, respectively.

**Value of the data**

● The data of the present study might increase the molecular information for *B. cereus* strains, isolated from crab in saline environment*.* The genomic data can be used in the development of other researchers such as comparative analyses using genes and genomes, origin and distribution of strains.
● Our data can give support for genetic engineering of *B. cereus* strains.
● This work can offer contributions for the molecular diversity of the *B. cereus* strains improving the pathogenicity assessment or probiotic application in the production of marine organisms.

## 1 Data

**Fig. 1:**
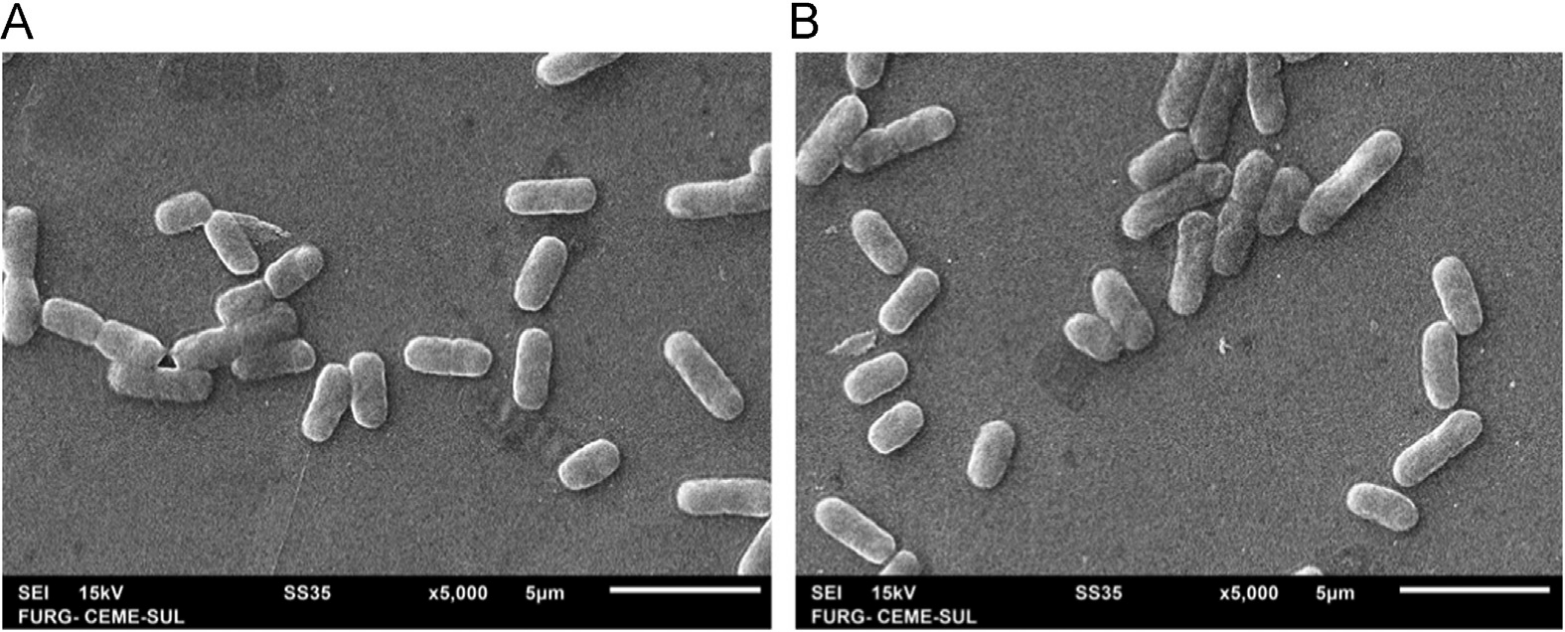
Scanning Electron Microscope of *Bacillus cereus* native strains #25 (A) and #29 (B).

**Fig. 2:**
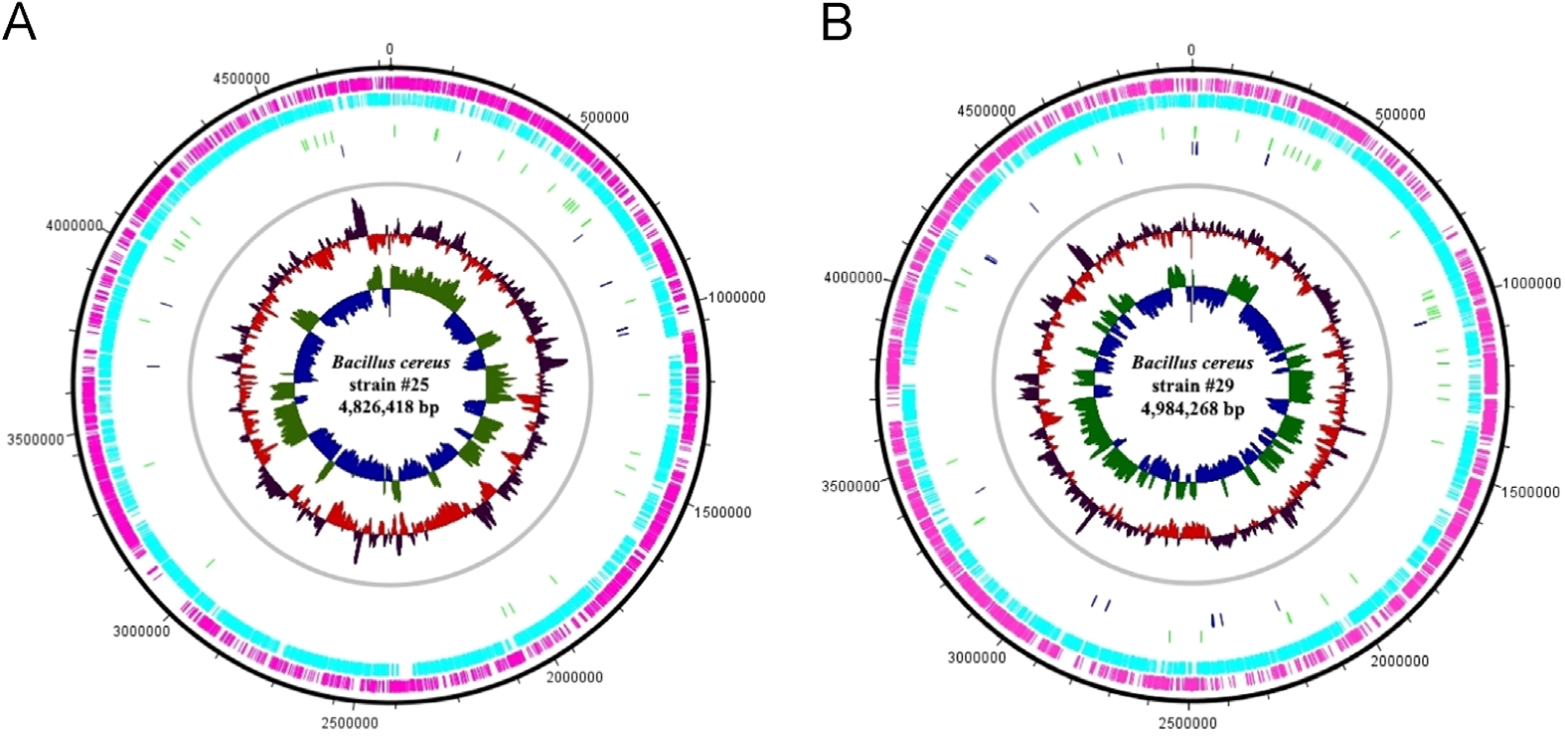
Chromosome features of native *Bacillus cereus* strains #25 (A) and #29 (B). Track 1, genome size. Tracks 2 and 3, coding sequence (CDS – forward and reverse). Tracks 4 and 5, genes and tRNA on forward and reverse strands. Track 6, chromosomal sequence. Tracks 7 and 8, GC content and GC skew [(G-C)/(G+C)].

In the present work, we describe the whole-genome shotgun (WGS) analysis of two strains of *B. cereus* (Fig. 1) from the collection of bacterial morphotypes from Laboratory of Environmental and Fish Microbiology at the Institute of Marine Sciences (LABOMAR/UFC – Brazil). The strains presented 99% of identity with the *Bacillus cereus* ATCC 14579 (accession number NC_004722). Genomic features of *Bacillus cereus* strains #25 and # 29 (Table 1) presented similar size, noncoding sequences, transfer RNA and ribosomal RNA sequences, respectively. Chromosome features of *Bacillus cereus* strains were drawing, as showed in Fig. 2.

**Table 1:** Genomic features of native *Bacillus cereus* strains #25 and # 29.

Strain	Genomic size	Gaps	C+G (%)	CDS	tRNAs	rRNAs
#25	4.82 Mb	66	35.11	5132	21	2
#29	4.98 Mb	161	35.32	5443	77	5

C+G (%): guanine and cytosine content; CDS: protein coding genes; tRNAs: transfer RNA; rRNAs: ribosomal RNA.

## 2 Experimental design, materials and methods

### 2.1 Isolation of native *Bacillus* strains

The native strains #25 and #29 were isolated from intestinal tract of the crab *Ucides* sp., from Pacoti River in the State of Ceará, Brazil. The intestinal tracts were homogenized with saline solution (NaCl 0.85%) and incubated at 70 °C for 1 h. An inoculum was plated on solid medium (NaCl 1.5%) and maintained at 30 °C for 48 h. The colonies similar with *Bacillus* were inoculated in Tryptone Soy Agar (TSA) and the culture integrity was evaluated with Gram Staining by microscopy. The gram positive *Bacillus* with sporulation capacity were destined to the Laboratory of Structural Genomics of the Federal University of Pelotas (UFPel, Brazil), for whole genome sequencing.

### 2.2 Isolation of genomic DNA

The strains were cultivated in Luria-Bertani (LB) medium at 37 °C with vigorous shaking (250 rpm) for 12 hours. The integrity was accessed by microscopy using Gram Staining method. Scanning Electron Microscope (JEOL JSM – 6610 LV) was performed using the pellet from culture washed twice with ultrapure water and quickly fixed in Bunsen flame. Genomic DNA was obtained using *Illustra Bacteria GenomicPrep Mini Spin* kit (GE Healthcare, USA), according the manufacturers guidelines.

### 2.3 Library preparation and sequencing

Bacterial genome sequencing was performed using the Ion Torrent PGM (Life Technologies, Saint Aubin, France) on 100 ng of DNA. The DNA library was constructed using enzymatic fragmentation and adaptor ligation with the Ion Xpress Plus fragment library kit (Life Technologies). Fragment size selection was performed using E-Gel® SizeSelect 2% (Invitrogen). After diluting the library at 100 pM, template preparation, emulsion PCR, and ion sphere particle (ISP) enrichment were performed using the Ion One Touch template kit (Life Technologies). The ISPs were loaded and sequenced on a 318 chip (Life Technologies).

### 2.4 Genome assembly

Raw sequence reads were obtained from the Ion Torrent server in BAM format and converted to FASTQ using the bamToFastq program from the BedTools package [1]. Reads with more than 20% of the bases with Phred score (Q) smaller than 20 where removed using the fastq_quality_filter script from the fastx-toolkit package (http://hannonlab.cshl.edu/fastx_toolkit/). *De novo* genome assemblies were generated for each strain using MIRA (http://mira-assembler.sourceforge.net/), SPAdes [2] and Newbler (http://www.roche.com/), and for each strain a consensus assembly was generated using CISA [3]. The final set of contigs from each strain was ordered with the program CAR [4], which is able to identify genome inversions and transpositions, using the genome of *Bacillus cereus* strain 03BB102 (GenBank: CP009318.1) as reference. The genome annotation was performed using Genix [5], and manually revised using Artemis [6].

A BLAST search [7] was performed against the NCBI nucleotide database (https://www.ncbi.nlm.nih.gov/BLAST/) to identify the most similar strain. Chromosome features of *Bacillus cereus* strains were drawing using DNAPlotter [8].
