# Supplementary material for: Complete genome sequence of native Bacillus cereus strains isolated from intestinal tract of the crab Ucides sp
Source: Data Brief. 2017 Nov 20;16:381–5. doi: 10.1016/j.dib.2017.11.049 (PMC5723261; doi:10.1016/j.dib.2017.11.049)
Supplement: Supplementary file 1 — Supplementary material [file mmc1.docx]

Dear Editor of Data in Brief

The authors declare no conflict of interest.

Luis Fernando Marins
